# Investigation and evaluation of cross-term reduction in masked Wigner-Ville distributions using S-transforms

**DOI:** 10.1371/journal.pone.0310721

**Published:** 2024-11-06

**Authors:** Nattapol Aunsri, Prasara Jakkaew, Chanin Kuptametee

**Affiliations:** 1 School of Applied Digital Technology, Mae Fah Luang University, Chiang Rai, Thailand; 2 Computer and Communication Engineering for Capacity Building Research Center, Mae Fah Luang University, Chiang Rai, Thailand; SRM Institute of Sciences and Technology, INDIA

## Abstract

Non-linear and non-stationary signals are analyzed and processed in the time-frequency (TF) domain due to interpretation simplicity. Wigner-Ville distribution (WVD) delivers a very sharp resolution of non-stationary signals in the TF domain. However, cross-terms occur between true frequency modes due to their bilinear nature. Masked WVD reduces cross-terms by multiplying the time-frequency representation (TFR) obtained from the WVD with the TFR of the same signal obtained from another method, while S-transform (ST) is a linear signal analysis method that combines the advantages of short-time Fourier transform (STFT) and wavelet transform (WT). This paper investigated WVD masking with both original and modified STs to compare their cross-term reduction results. Moreover, additional parameters are integrated into the ST to deliver the better resolution of the ST and, consequently, more satisfactory cross-term reduction. However, these parameters must be carefully optimized by expert users in their respective application fields.

## Section 1: Introduction

Most signals in real-world problems and applications are non-stationary. The number of frequency components (or frequency modes for some applications) can vary with time, while the relationship between frequencies and time is non-linear [1]. Therefore, non-stationary signals are analyzed and processed in the time-frequency domain using time-frequency distribution (TFD) methods in practical applications [2–18].

Many methods have been proposed to obtain TFDs of non-stationary signals. Short-time Fourier transform (STFT) is a linear method that performs FT on the signal multiplied by a sliding real and even window function that localizes the spectrum in time. STFT can be easily implemented but the length of the window function must be carefully considered. A longer window yields better frequency resolution while a shorter window yields better time resolution [19]. Furthermore, the chosen type of window function also affects the time-frequency representation (TFR) of the signal [10].

Wavelet transform (WT) employs a basis of orthogonal functions that have already been localized in time to locally expands and contracts the input signal with frequency in order to represent the local features of the input signal [19]. Wavelet functions have different irregular shapes to be chosen to analyze discontinuities in the signal and WT can be applied in many research works [20–23]. In [20], Haar wavelets were applied in the finite volume (FV) method to study shallow water surfaces. Work performed in [21] assesses the quality of the images using wavelet decomposition. In addition, presented in [22], this work applied Haar wavelet collocation methods (HWCMs) to approximate the quantum wave equation named Kelin-Gordon equation. Furthermore empirical wavelet transform (EWT) was employed to improve medical image classification. WT can provide variable resolutions for the TFR of the signal, while STFT cannot [23]. However, WT does not retain phase information while STFT does [19]. S-transform (or Stockwell transform, ST) [24] was first proposed in 1996. This has advantages over STFT and WT because ST retains phase information and uses a Gaussian window function whose the length depends on the frequency value of the signal [19, 24]. However, the frequency-axis resolution gets poorer for the high-frequency components while the time-axis resolution gets poorer for the low-frequency components. Modified STs with more adaptive or optimized window functions then have been proposed to obtain TFRs with better resolutions [25–29].

The Wigner-Ville distribution (WVD) method provides TFR of a signal with a very sharp resolution. However, its bilinear nature causes false frequency components called “cross-terms” that occur between the true components (or auto-terms) of the TFR. These cross-terms are unwanted in signal analysis and processing because they interfere with TFR interpretation [30], resulting in ambiguity and difficulty in signal separation problems. A simple idea for mitigating the cross-terms is to decompose the input signal into several signal components and the WVD of each component is found before we add these WVDs together to obtain the cross-term free WVD. The signals with a few frequency components that are not very complicated can be decomposed employing, for examples, empirical mode decomposition (EMD) and variational mode decomposition (VMD), in order to ensure the effectiveness of cross-term reduction as shown in, for example, Refs. [31–35]. However, in fact, each extracted component may not always have a constant frequency value or a chirp frequency mode (i.e., a frequency mode that varies linearly with time) and they depend on the selected decomposition method. For this reason, the WVD method has been widely studied for further improvements [36–45].

A masked WVD of any signal can be obtained by performing elementwise multiplication between its original WVD and its cross-term-free TFR such as its spectrogram (obtained by using STFT) [30, 46]. Although the procedure of this data-driven technique seems simple, the resolution of the masked WVD actually gets better since the magnitudes of the WVD at the TF coordinates where the cross-terms exist are multiplied by the near-zero magnitudes of the cross-term free TFR, and, consequently, the cross-terms are mitigated. Ref. [47] applies the smoothed pseudo WVD to design the filters used for masking the WVD to obtain the TFR with mitigated cross-terms. However, the choices of masking filters must be carefully chosen according to the applications. Since ST is another linear cross-term free TFD method that is superior than STFT and are studied widely for improving its delivered resolution, this paper then investigated the effects of using standard and modified STs to reduce cross-terms in WVDs.

The structure of this paper is as follows. Section 2: Wigner-Vill distribution and Section 3: S-transform provide the details about WVD and ST, respectively. Section 4: Simulation results shows results of masking WVDs of non-stationary signals with some state-of-the-art STs and Section 6: Conclusions draws conclusions of this work.

## Section 2: Wigner-Ville distribution

Wigner-Ville distribution is a bilinear (or quadratic) signal analysis method that finds the time-frequency representation (TFR) of the input signal *x*(*t*) as [19, 48]

$$\begin{array}{c} W_{x}\left(t,f\right)=\int_{-\infty }^{\infty }x_{a}\left(t+\frac{\tau }{2}\right)x_{a}^{*}\left(t-\frac{\tau }{2}\right)e^{-j2\pi f\tau }d\tau \end{array}$$ (1)

where

$$\begin{array}{c} x_{a}\left(t\right)=x\left(t\right)+jH\left\{x\left(t\right)\right\} \end{array}$$ (2)

is the signal to be analyzed with WVD found from *x*(*t*), $x_{a}^{*}\left(t\right)$ is the complex conjugate of *x*_*a*_(*t*), and H{x(t)} represents the Hilbert transform of *x*(*t*).

Since WVD is bilinear, if there are many components in the signal (which can be true signal components or noises), cross-terms always occur between signal components. Cross-terms are useful in classification applications but they need to be eliminated or at least reduced since they cause difficulties in analyzing non-stationary signals in the time-frequency domain, especially if true components are close to each other [30, 33]. The WVDs of mono-component non-linear signals also face this problem [30, 43]. A simple technique to reduce cross-terms in WVD is to multiply the WVD of a signal with its spectrogram (which is the squared magnitude of STFT of the signal) [19, 30, 46]. The resolution of TFR obtained by spectrogram is poorer than WVD but it has no cross-terms [19]. The multiplication suppresses the intensities of the areas of TFR where cross-terms locate. The resulting TFR obtained from this technique is called masked WVD [46]. Its resolution is sharper than the spectrogram and its TFR is cleaner than the original WVD [30].

## Section 3: S-transform

S-transform (or Stockwell transform, ST) [24] is a linear signal analysis method considered equivalent to frequency-dependent STFT or phase-corrected WT as [19, 49]

$$\begin{array}{c} ST_{x}\left(t,f\right)=e^{-j2\pi ft}\Psi_{x}^{\psi }\left(t,f\right) \end{array}$$ (3)

where

$$\begin{array}{c} \Psi_{x}^{\psi }\left(t,f\right)=\int_{-\infty }^{\infty }x\left(\tau \right)\psi \left(\tau -t,f\right)d\tau \end{array}$$ (4)

is the WT of the signal *x*(*t*), *e*^−*j*2*πft*^ is the phase correction factor, and *ψ*(*t*, *f*) is the mother wavelet defined as

$$\begin{array}{c} \psi \left(t,f\right)=\frac{\left|f\right|}{\sqrt{2\pi }}e^{\frac{-t^{2}f^{2}}{2}}e^{-j2\pi ft}. \end{array}$$ (5)

The ST can be expressed as

$$\begin{array}{c} ST_{x}(t,f)=e^{-j2\pi ft}\int_{-\infty }^{\infty }x(\tau )\frac{\left|f\right|}{\sqrt{2\pi }}e^{\frac{-{\left(\tau -t\right)}^{2}f^{2}}{2}}e^{-j2\pi f(\tau -t)}d\tau \\ ST_{x}(t,f)=\int_{-\infty }^{\infty }x(\tau )\frac{\left|f\right|}{\sqrt{2\pi }}e^{\frac{-{\left(\tau -t\right)}^{2}f^{2}}{2}}e^{-j2\pi f\tau }d\tau . \end{array}$$ (6)

and generalized as [19, 27, 49]

$$\begin{array}{c} ST_{x}\left(t,f,\sigma_{f}\right)=\int_{-\infty }^{\infty }x\left(\tau \right)w\left(\tau -t,\sigma_{f}\right)e^{-j2\pi f\tau }d\tau \end{array}$$ (7)

where

$$\begin{array}{c} w\left(\tau -t,\sigma_{f}\right)=\frac{1}{\sqrt{2\pi }\sigma_{f}}e^{\frac{-{\left(\tau -t\right)}^{2}}{2\sigma_{f}^{2}}} \end{array}$$ (8)

is the Gaussian window function, and *σ*_*f*_ is the standard deviation, portraying the width of the Gaussian window, as a function of frequency *f*. That is, the original ST [24] employs a window function with variable lengths and simply sets *σ*_*f*_ = |*f*|^−1^. However, the original ST cannot represent high-frequency components with satisfactory frequency-axis resolution. The modifications of ST then were made, e.g., Refs. [25–27, 50, 51].

Ref. [25] adjusted the window’s width with by using *σ*_*f*_ = |*f*|^−*r*^. For *f* > 1 Hz, if 0 < *r* < 1, the window gets wider in the time-domain, while the window becomes narrower if *r* > 1. The improved generalized ST (IGST) [51] expands the generalization of the *σ*_*f*_ as:

$$\begin{array}{c} \sigma_{f}=\frac{1}{m{\left(\left|f+p\right|\right)}^{r}}, \end{array}$$ (9)

where the parameters *m* ∈ (0, 3], *p* ∈ [0, 3], and *r* ∈ [0, 1] have to be set to satisfy the conditions

$$\begin{array}{c} \alpha T_{s}m{\left(f_{\text{max}}+p\right)}^{r}-1\leq 0 \end{array}$$ (10)

and

$$\begin{array}{c} 1-\beta T_{s}m{\left(f_{\text{min}}+p\right)}^{r}\leq 0 \end{array}$$ (11)

where *T*_*s*_ = 1/*f*_*s*_ is the sampling period, *f*_min_ = 1 Hz, *f*_max_ = *f*_*s*_/2 Hz, *α* = 10, and *β* = 1, 000. Let *P* = {*m*, *p*, *r*} denotes the set of parameters used to optimize the TFR, where its members can be found according to the energy concentration measure (CM) [25, 52] as

$$\begin{array}{c} P_{\text{opt}}=\text{arg}\;\underset{P}{\text{max}}\left(CM_{P}\right), \end{array}$$ (12)

where

$$\begin{array}{c} CM_{P}=\frac{1}{\int_{-\infty }^{\infty }\int_{-\infty }^{\infty }\left|\bar{TFR_{x}^{P}\left(t,f,\sigma_{f}\right)}\right|dtdf} \end{array}$$ (13)

and

$$\begin{array}{c} \bar{TFR_{x}^{P}\left(t,f,\sigma_{f}\right)}=\frac{TFR_{x}^{P}\left(t,f,\sigma_{f}\right)}{\sqrt{\int_{-\infty }^{\infty }\int_{-\infty }^{\infty }{\left|TFR_{x}^{P}\left(t,f,\sigma_{f}\right)\right|}^{2}dtdf}} \end{array}$$ (14)

is the module of the TFR at the TF-coordinate (*t*, *f*) with normalized energy and *σ*_*f*_ that is found using parameters in *P*_opt_. That is, IGST [51] focuses on finding parameters that maximize CM of the TFR of the ST.

The input-driven ST called modified ST (MST) [26] proposed the input signal-dependent *σ*_*f*_ as:

$$\begin{array}{c} \sigma_{f}=\frac{\left(f/L_{x}\right)+\gamma \sigma_{x}^{2}}{\left|f\right|}, \end{array}$$ (15)

where *L*_*x*_ and $\sigma_{x}^{2}$ are the length and the variance of amplitudes of *L*_*x*_ data samples of the time-domain input signal *x*(*t*), respectively. The parameter *γ* was manually set according to the input signal to be analyzed. Ref. [53], however, proposed the compact support kernel (CSK) to replace the Gaussian window and Ref. [54] proposed the linear canonical ST (LCST) which is based on the specific convolution theorem in linear canonical transform (LCT) domain. They were proved to enhance the quality of TFR signals but they required additional parameters carefully configured for each signal.

## Section 4: Simulation results

**Fig 1:**
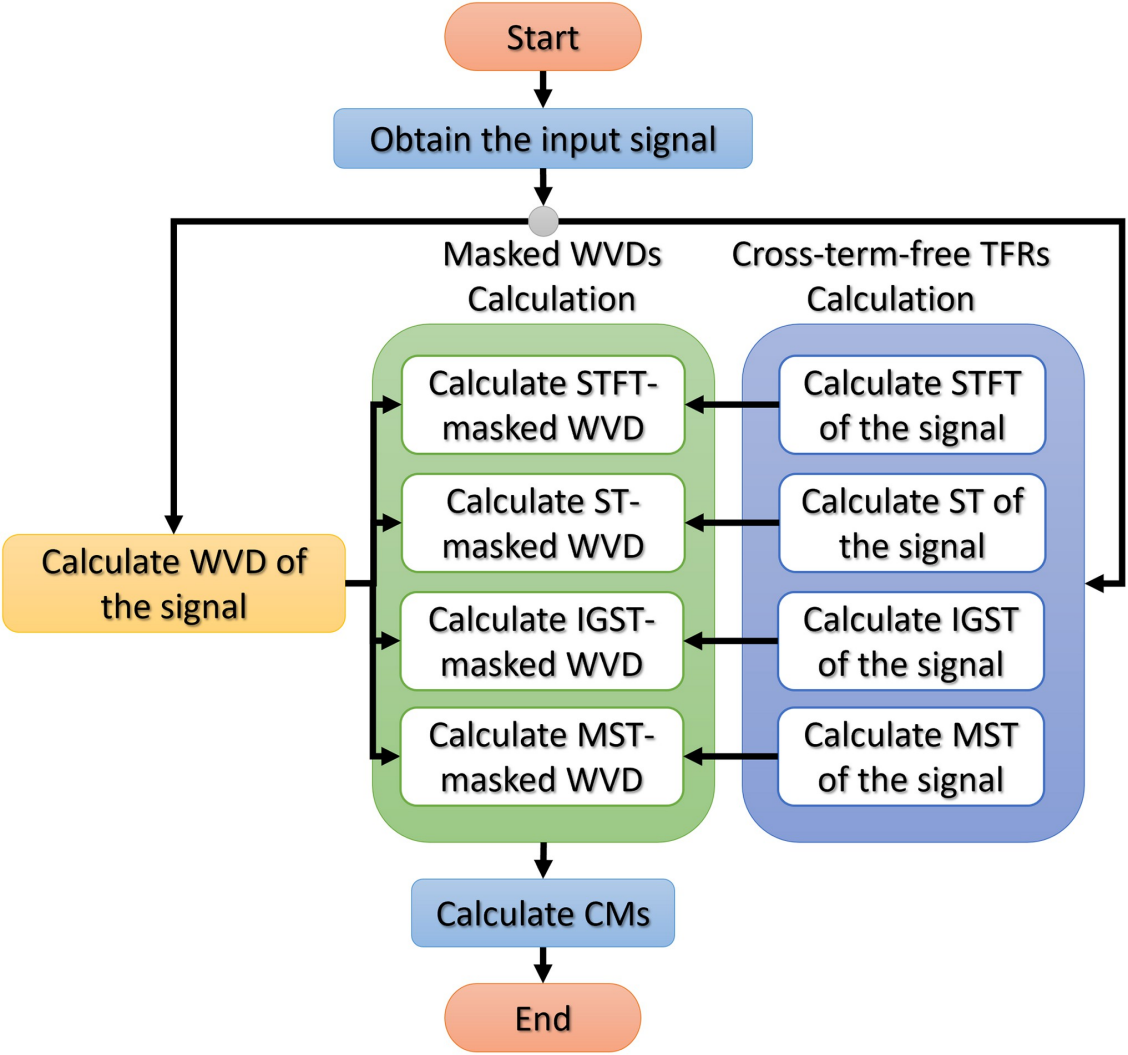
The flowchart of the process of conducting the investigation study.

This paper investigated how modifications of the Gaussian window function used in STs impacted the resolution of masked WVDs. For each signal presented in this section, we used the spectrogram obtained from STFTs with a fixed-length Gaussian window at the length of 101 data samples to mask WVDs to obtain fair comparison since STs also use Gaussian windows but with adaptive lengths. The improved generalized ST (IGST) [51] and the modified ST (MST) [26] are chosen to compare their results with STFT-masked and original ST-masked WVDs. The overall process of the investigation study in this paper is presented in Fig 1.

We employ two metrics to evaluate concentration of the TFRs. First, we calculate the energy CM of each TFR which employs Eqs 13 and 14. Higher CMs mean higher concentration. Second, we calculate Rényi entropy (RE) [55] of each TFR which can be found as

$$\begin{array}{c} RE_{\epsilon }=\frac{1}{1-\epsilon }{\text{log}}_{2}\int_{-\infty }^{\infty }\int_{-\infty }^{\infty }{\left|\bar{TFR_{x}\left(t,f,\sigma_{f}\right)}\right|}^{\epsilon }dtdf, \end{array}$$ (16)

where *ϵ* ≥ 3 is the recommended order number of RE. We, in this paper, select *ϵ* = 3 because this value is commonly employed for most of signals and their TFRs [56–58]. However, as opposed to CMs, lower REs denote higher concentration. Note that, in case of any discrete TFR, we must first re-normalize value of each module $\bar{TFR_{x}\left(t,f,\sigma_{f}\right)}$ (that is found using Eq 14 to ensure the unity summation.

### Synthetic signals

**Fig 2:**
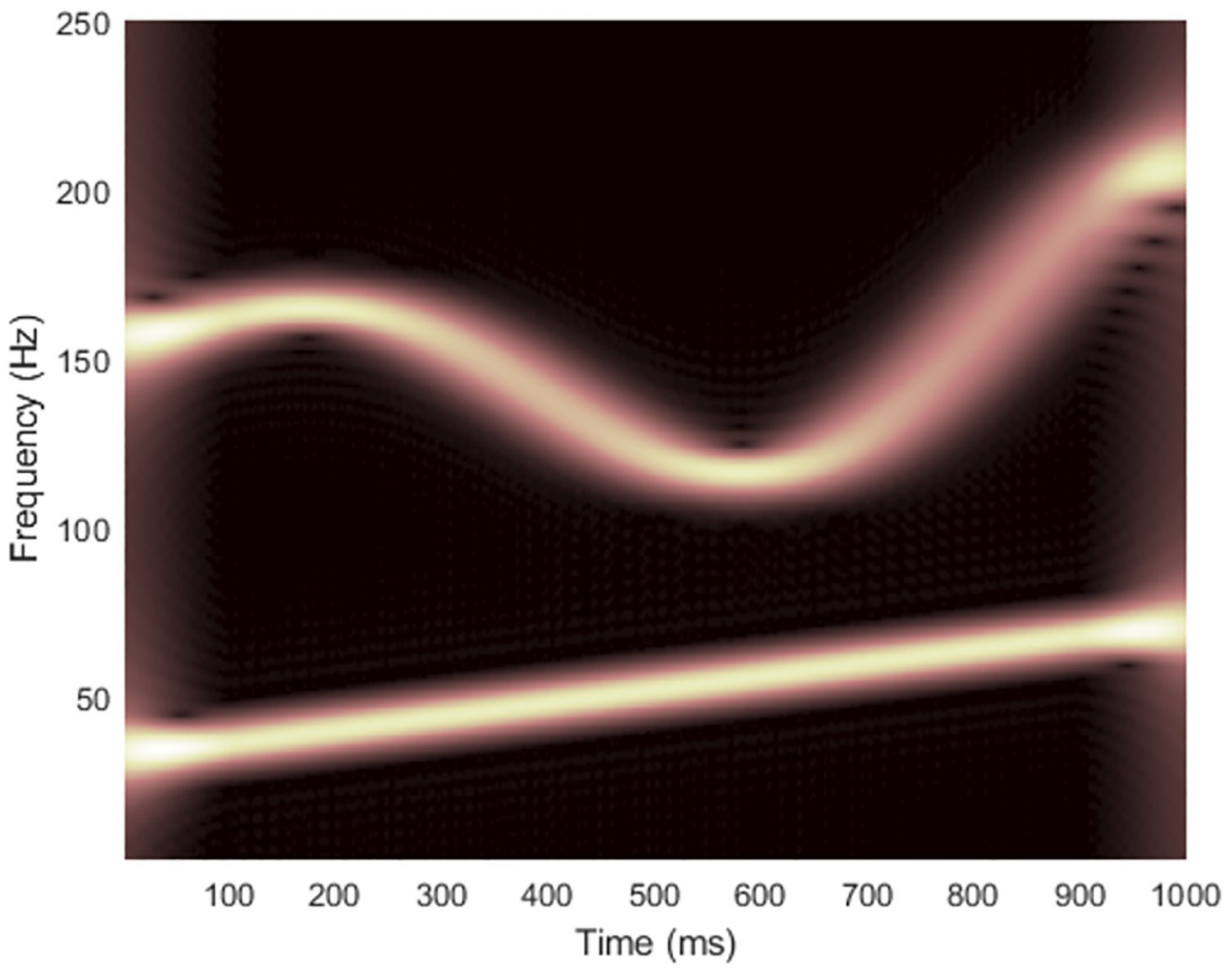
Spectrogram of the clean signal 1.

**Fig 3:**
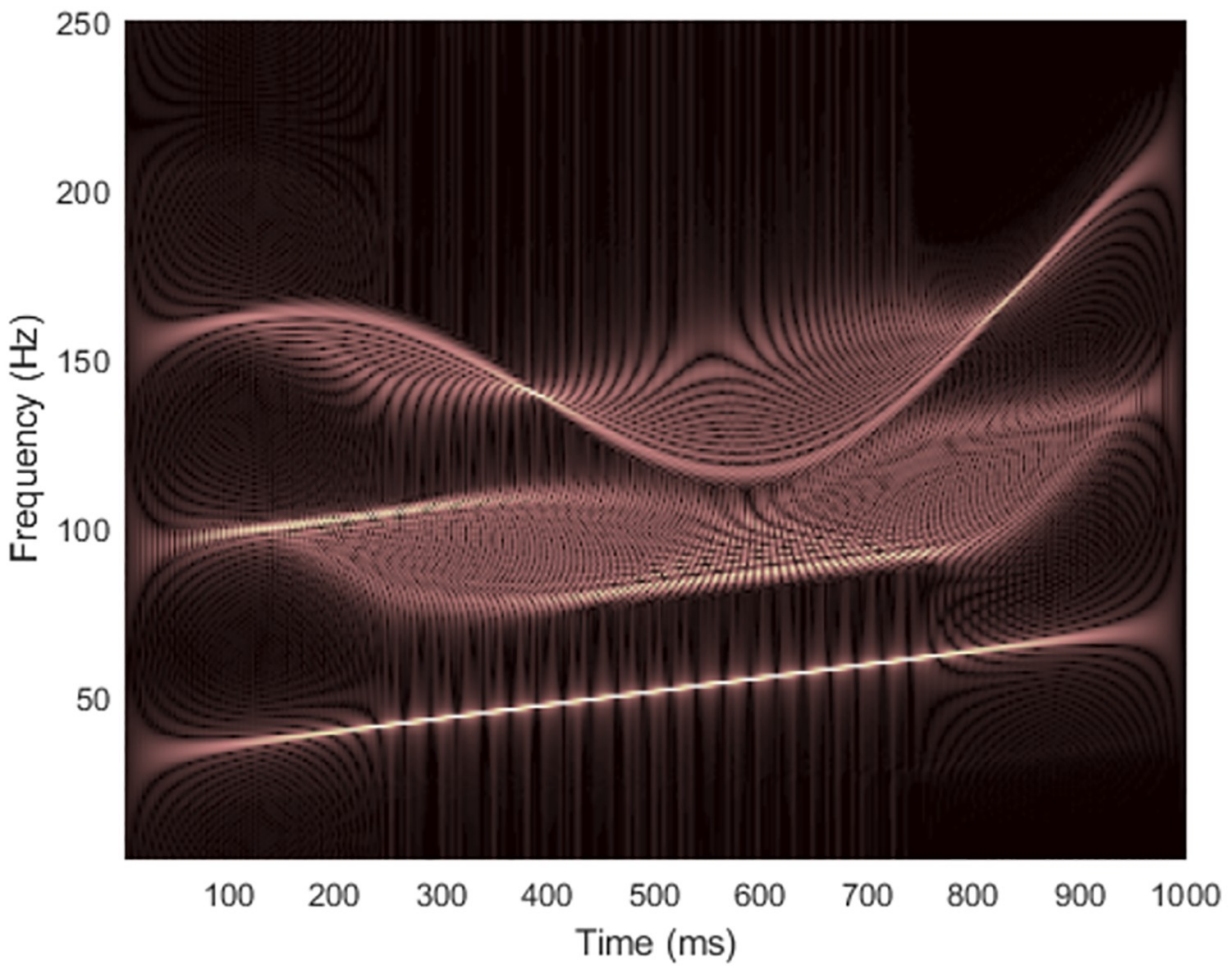
WVD of the clean signal 1.

We first start from investigating masking WVD of a simple synthesized signal (called signal 1). This signal includes a linear low-frequency component and a non-linear high-frequency component as shown by the equation:

$$\begin{array}{c} x_{S1}\left(t\right)=\text{cos}\left[2\pi (20t+30)t\right]+\text{cos}\left[2\pi (10\text{sin}\left(2\pi t\right)+150)t\right], \end{array}$$ (17)

where the signal starts at time *t* = 0, the sampling rate is *f*_*s*_ = 500 Hz and the number of data samples is *L*_*x*_ = 500. Figs 2 and 3 show the spectrogram and WVD of a clean synthesized non-stationary signal with the matrix dimension at (*f*_*s*_/2) × *L*_*x*_ = 250 × 500.

**Fig 4:**
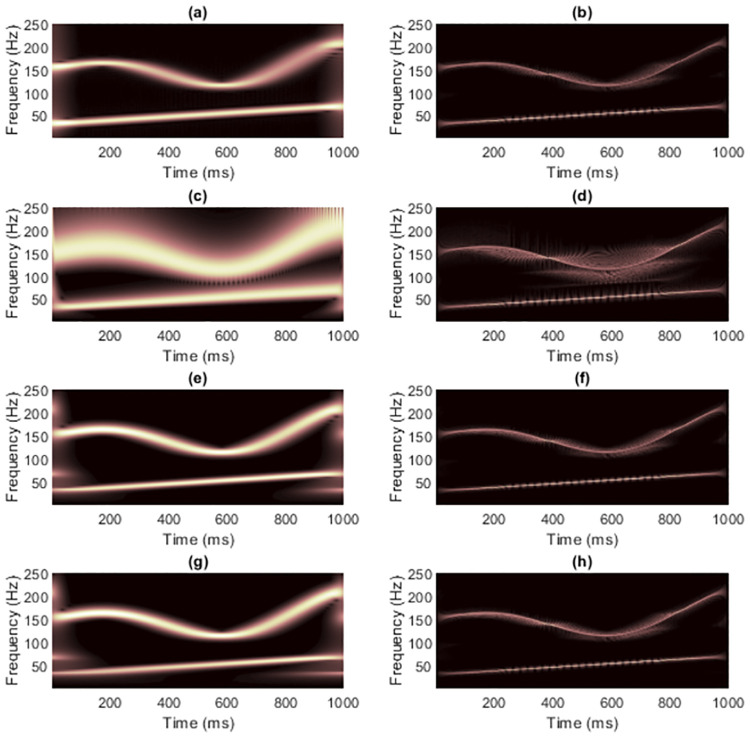
TFRs of the clean signal 1 obtained from (a) STFT, (b) the STFT-masked WVD, (c) the original ST [24], (d) the ST-masked WVD, (e) the IGST [51] with *P*_opt_ = {1.0452, 0.9908, 0.6956}, (f) the IGST-masked WVD, (g) the MST [26] with *γ* = 4 and (h) the MST-masked WVD.

Fig 4 shows the TFRs of signal 1 obtained from non-quadratic methods (i.e., STFT and STs, on the left-hand side) along with the results of masking WVD of the clean signal (on the right-hand side). Because this signal has with few components and long enough length. Using STFT then seemed to be sufficient to mask WVD, while the window length required fixing carefully. Masking the WVD with the original ST [24] was least effective compared to the others, even to the spectrogram, since the resolution of the non-linear frequency component in Fig 4(c) clearly spread across the area of high-frequency values. High-frequency cross-terms then were not successfully masked. The ST-masked WVDs could be improved if optimization techniques were first applied to the STs. However, TFRs delivered by IGST [51] and MST [26] had low-frequency energies that spread among the time-axis at the beginning and at the end in the time-domain. That is, MST [26] and IGST [51] faced the problems of resolution at the area of low-frequency values. Problem in the MST [26] was worse than that in the IGST [51] since the low-frequency at the end of the signal spread wider (at around 600-1,000 ms compared to the IGST [51] at around 800-1,000 ms). No other significant differences were seen between the WVDs that were masked by these two modified STs.

**Fig 5:**
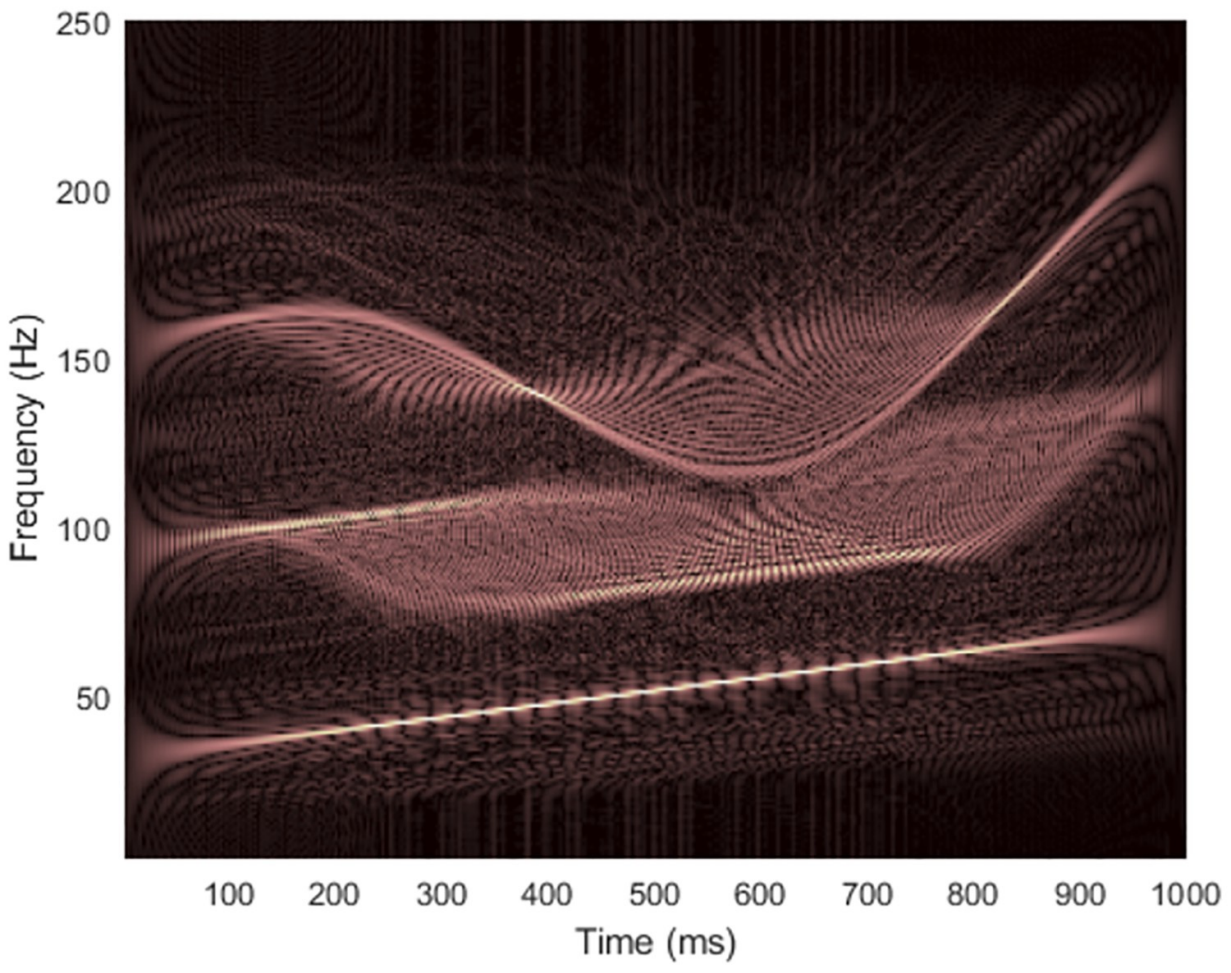
WVD of the noisy signal 1 at SNR 15 dB.

**Fig 6:**
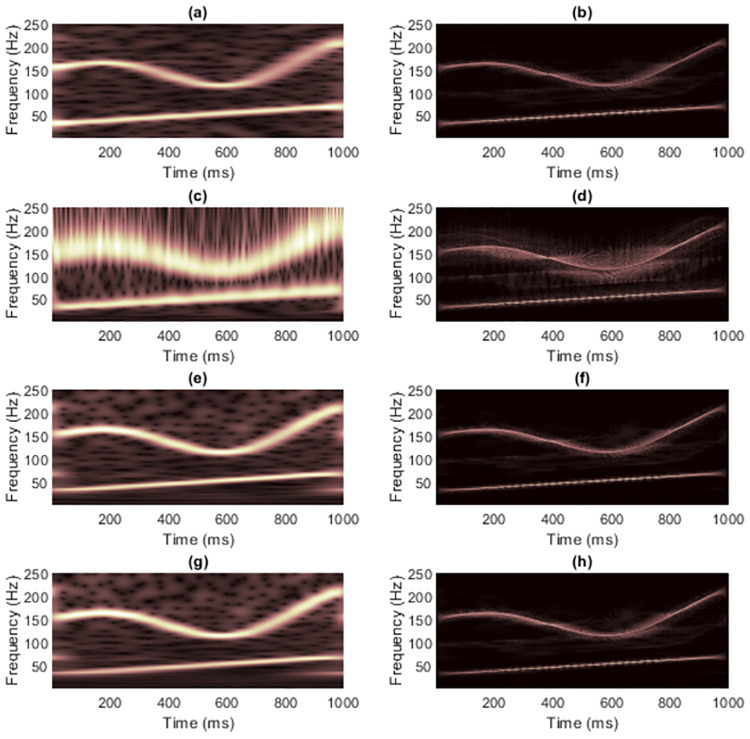
TFRs of the noisy signal 1 at SNR 15 dB obtained from (a) STFT, (b) the STFT-masked WVD, (c) the original ST [24], (d) the ST-masked WVD, (e) the IGST [51] with *P*_opt_ = {1.0314, 0.9910, 0.6900}, (f) the IGST-masked WVD, (g) the MST [26] with *γ* = 4 and (h) the MST-masked WVD.

Masking was then performed on WVDs of signal 1, now contaminated with an additive white Gaussian noise (AWGN) in time-domain at the signal-to-noise ratio (SNR) of 15 dB (shown in Fig 5), with results displayed in Fig 6. Low-frequency energies of the added noise in TFRs delivered by STs were sharper than in the spectrograms, so low-frequency energies were more suppressed, especially in modified STs compared to the STFT. However, energies of high-frequency noise appearing in TFRs of IGST and MST seemed to be denser than that appearing in the spectrogram. In this case, the cross-terms that occurred between two true components were not suppressed easily due to the added noise. Note that characteristics of the added time-domain Gaussian noise when represented in TF-domain were no longer Gaussian [15].

Results of masking WVD of the signal 1 at different SNRs with different non-quadratic methods were also provided in terms of CMs and REs in Tables 1 and 2, respectively. Original ST was again proved being least efficient to be employed to mask the WVD because, at each SNR, concentration of the TFR delivered by the original ST was lower than concentrations of the TFRs delivered by other non-quadratic methods by having lowest CM and highest RE. Also, the original ST-masked WVD had the lowest CM and the highest RE compared to those of other masked WVDs. While difference between the resolution of IGST-masked WVD and MST-masked WVD could not be clearly seen, CMs and REs of IGST-masked WVDs were slightly higher than those of MST-masked WVDs at each SNR. For case of clean signal 1, while CM of its IGST was higher than CM of its spectrogram, CM of its IGST-masked WVD was lower than CM of its STFT-masked WVD. REs, however, show consistency that RE of a masked WVD would be low, if RE of TFR delivered by that employed non-quadratic was also low. RE of spectrogram was lower than those of TFRs delivered from other non-quadratic methods at the same SNR and RE of STFT-masked was lower than those of the other masked WVDs at the same SNR.

**Table 1:** Comparisons of CMs of TFRs of the synthesized signal 1.

SNR (dB)	WVD (×10^−3^)	Masking TFR		Masked WVD (×10^−3^)
		Method	CM(×10^−3^)	
Clean	6.0669	STFT	6.5213	**13.3897**
		ST	4.0552	8.6043
		IGST	**6.5464**	12.7876
		MST	6.4795	12.4150
15	5.5584	STFT	**5.3472**	**11.9371**
		ST	3.8636	7.7379
		IGST	5.2760	11.3803
		MST	5.2301	11.0296
10	5.0892	STFT	**4.6822**	**10.4080**
		ST	3.7094	6.9039
		IGST	4.6309	9.9829
		MST	4.5943	9.6525
5	4.5410	STFT	**3.9865**	**7.9777**
		ST	3.5337	5.8142
		IGST	3.9803	7.7551
		MST	3.9484	7.4559

**Table 2:** Comparisons of REs (at *ϵ* = 3) of TFRs of the synthesized signal 1.

SNR (dB)	WVD	Masking TFR		Masked WVD
		Method	RE	
Clean	14.3097	STFT	**14.3507**	**11.9472**
		ST	15.7824	13.1988
		IGST	14.3583	12.1474
		MST	14.3777	12.2637
15	14.5488	STFT	**14.8093**	**12.2343**
		ST	15.8990	13.4870
		IGST	14.8511	12.4368
		MST	14.8687	12.5567
10	14.8158	STFT	**15.1488**	**12.5822**
		ST	16.0049	13.8166
		IGST	15.1864	12.7677
		MST	15.2030	12.8933
5	15.2106	STFT	**15.6195**	**13.2989**
		ST	16.1373	14.3688
		IGST	15.6352	13.4395
		MST	15.6537	13.5723

**Fig 7:**
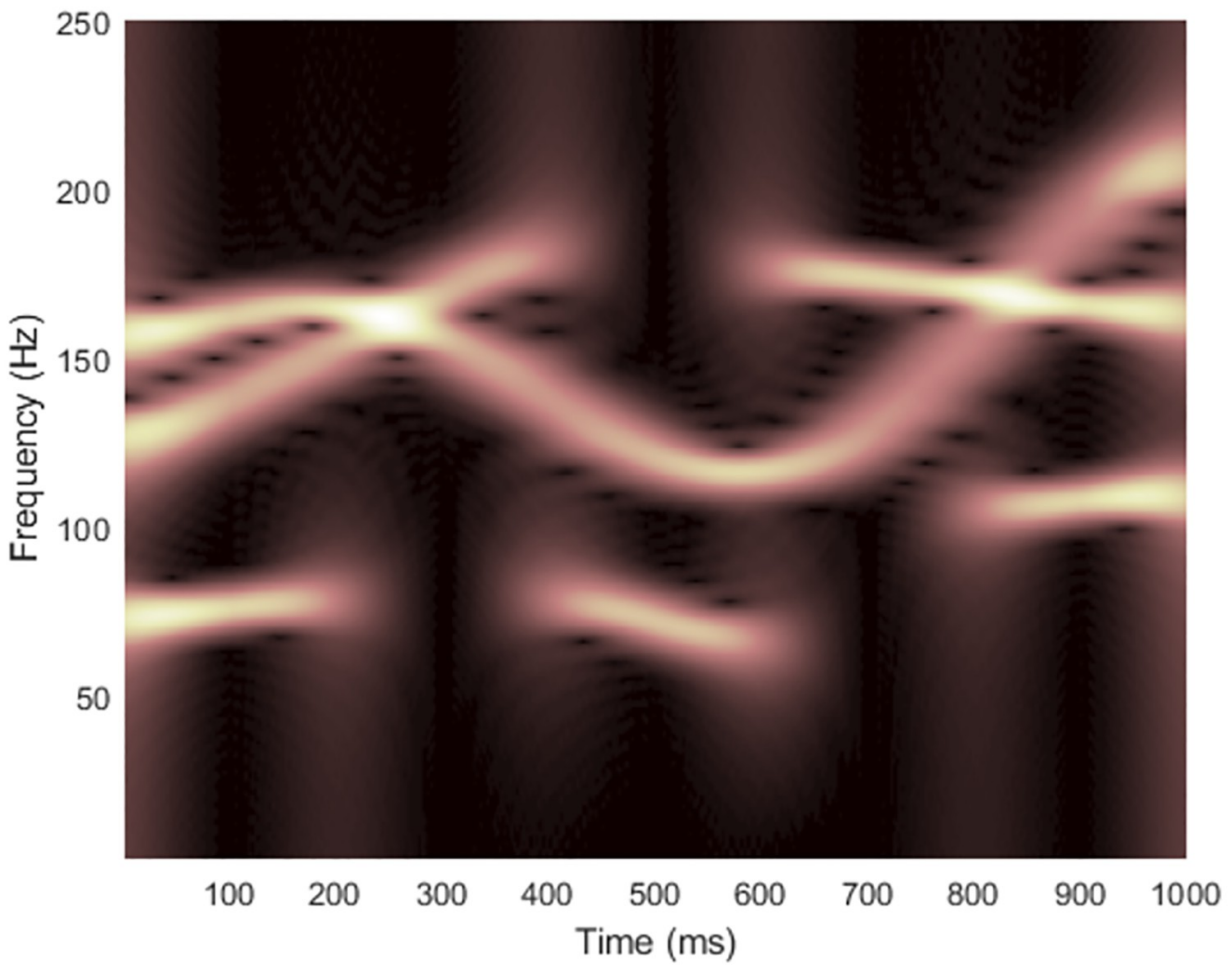
Spectrogram of the clean signal 2.

**Fig 8:**
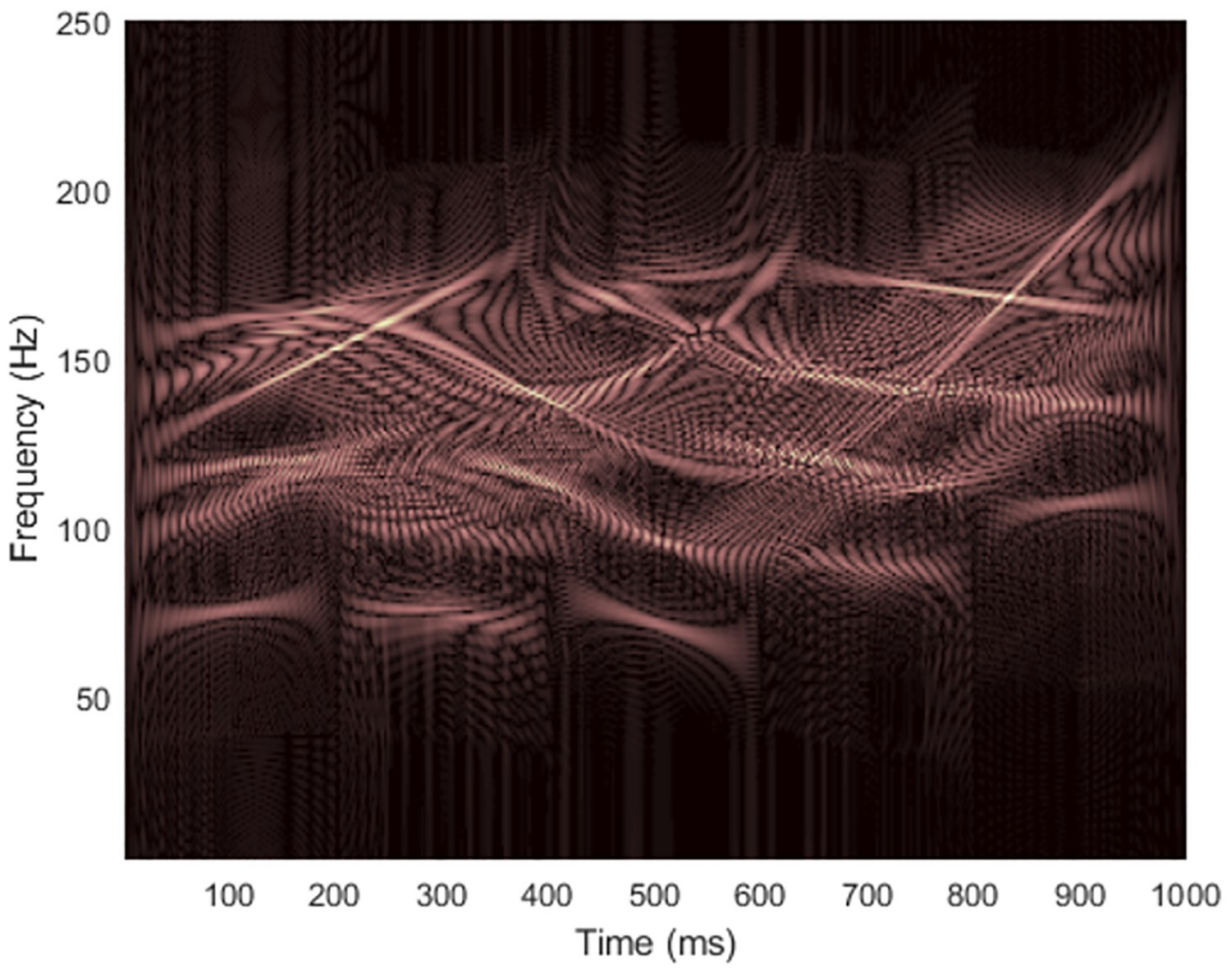
WVD of the clean signal 2.

To further illustrate the benefit of the masking technique, we also performed experiments on another synthetic signal (called signal 2). This signal consists of a constantly appearing frequency component $x_{S2}^{const}\left(t\right)=\text{cos}\left[2\pi (10\text{sin}\left(2\pi t\right)+150)t\right]$ and other frequency components that do not constantly appear as:

$$\begin{array}{c} x_{S2}\left(t\right)=\{\begin{array}{cc} x_{S2}^{const}\left(t\right)+\text{cos}\left[2\pi (80t+120)t\right]+\text{cos}\left[2\pi (20t+70)t\right], & \text{if}\;t\in [0,0.2)\\ x_{S2}^{const}\left(t\right)+\text{cos}\left[2\pi (80t+120)t\right], & \text{if}\;t\in [0.2,0.4)\\ x_{S2}^{const}\left(t\right)+\text{cos}\left[2\pi (-50t+120)t\right], & \text{if}\;t\in [0.4,0.6)\\ x_{S2}^{const}\left(t\right)+\text{cos}\left[2\pi (-20t+200)t\right], & \text{if}\;t\in [0.6,0.8)\\ x_{S2}^{const}\left(t\right)+\text{cos}\left[2\pi (-20t+200)t\right]+\text{cos}\left[2\pi (20t+70)t\right] & \text{if}\;t\in [0.8,1) \end{array}, \end{array}$$ (18)

where the signal starts at time *t* = 0, the sampling rate is *f*_*s*_ = 500 Hz and the number of data samples is *L*_*x*_ = 500. The clean spectrogram and the clean WVD are shown in Figs 7 and 8, respectively, with the TFR matrix dimension at (*f*_*s*_/2) × *L*_*x*_ = 250 × 500. This signal, however, contained more frequency components and some crossed each other, while each component was located closer to one other. This scenario was more complex compared to signal 1.

**Fig 9:**
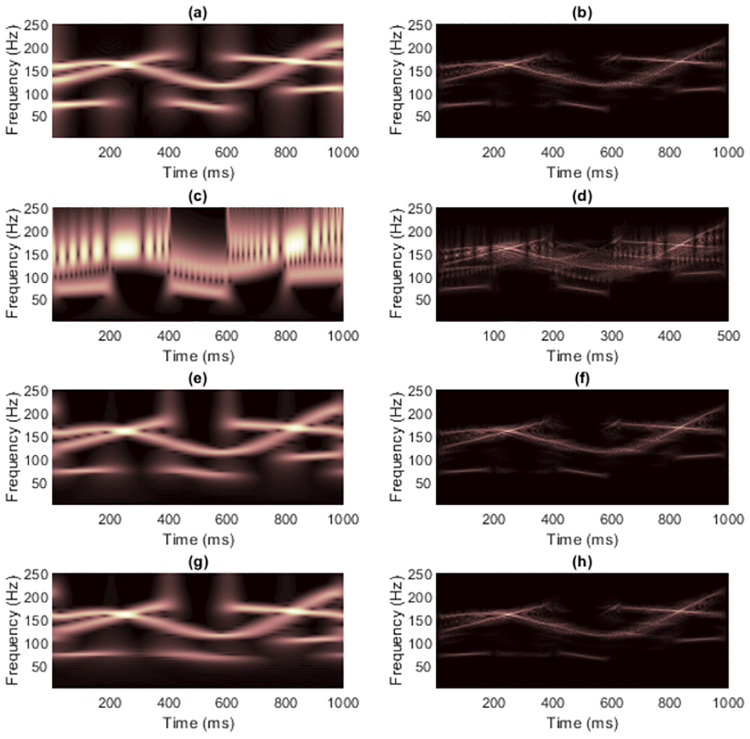
TFRs of the clean signal 2 obtained from (a) STFT, (b) the STFT-masked WVD, (c) the original ST [24], (d) the ST-masked WVD, (e) the IGST [51] with *P*_opt_ = {1.0063, 0.9914, 0.6979}, (f) the IGST-masked WVD, (g) the MST [26] with *γ* = 4 and (h) the MST-masked WVD.

**Fig 10:**
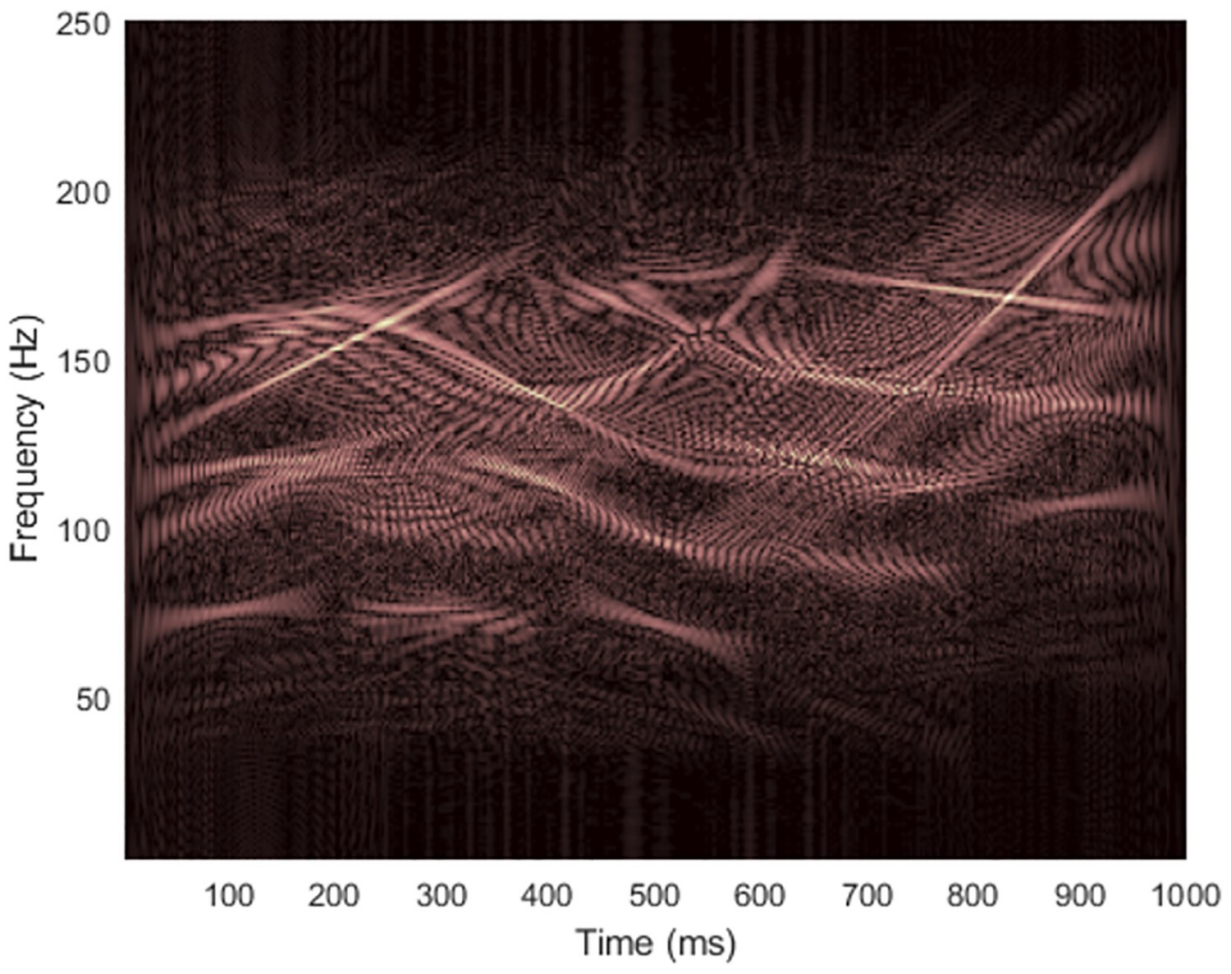
WVD of the noisy signal 2 at SNR 15 dB.

**Fig 11:**
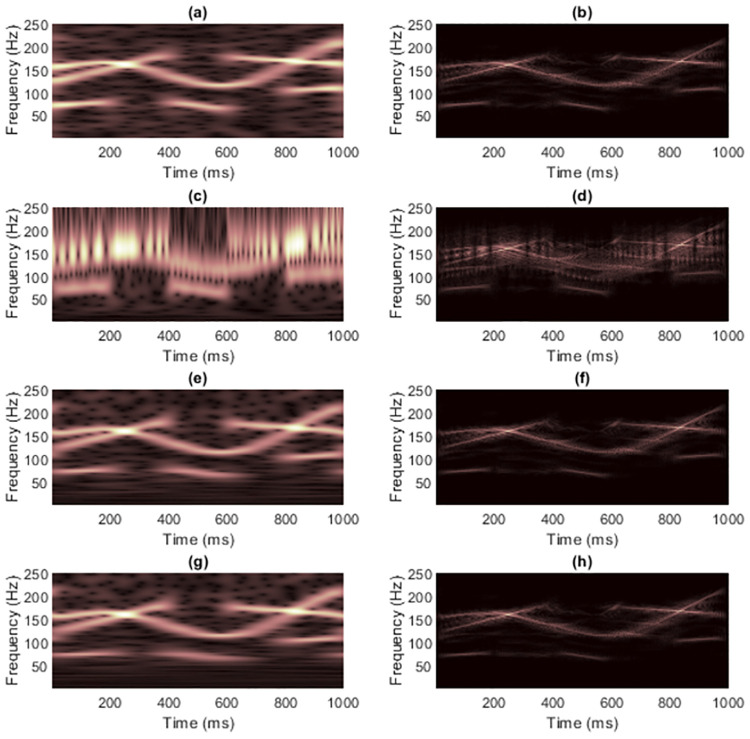
TFRs of the noisy signal 2 at the SNR 15 dB obtained from (a) STFT, (b) the STFT-masked WVD, (c) the original ST [24], (d) the ST-masked WVD, (e) the IGST [51] with *P*_opt_ = {1.0052, 0.9916, 0.6818}, (f) the IGST-masked WVD, (g) the MST [26] with *γ* = 4 and (h) the MST-masked WVD.

Fig 9 shows TFRs of signal 2 obtained from non-quadratic methods (i.e., STFT and STs, on the left-hand side) along with the results of WVD masking (on the right-hand side) in case the signal was clean. For this signal, resolution of the STFT-masked WVD was better than those of other masked WVDs at each SNR. The original ST still provided the worst overall quality of masked WVDs compared to the others, especially for high-frequency components where discontinuities across time existed. However, the low-frequency cross-terms could be attenuated more satisfactorily because low-frequency components in the TFR delivered by original ST did not spread much across time. IGST and MST delivered high-frequency energies with acceptably good resolution but they still faced problem of resolution of low-frequency energies. Consequently, they less successfully attenuated low-frequency cross-term located at time between 200 and 400 ms. The results of masking WVD of signal 2 that was corrupted with AWGN in time-domain at SNR 15 dB (shown in Fig 10) were also provided in Fig 11. In this case, performance in suppressing cross-terms was not surprisingly reduced.

Tables 3 and 4 respectively present comparisons of CMs and REs of the original WVDs, the non-quadratic TFRs used for WVD masking, masked WVDs and different SNRs of the signal 2 when AWGNs were added. While original ST still yielded the worst results, IGST and MST achieved the results that were superior to those of STFT by having greater CMs and lower REs. In case of clean signal 2, CM of TFR delivered by IGST was higher than CM of MST, but CM of IGST-masked WVD was lower than CM of MST-masked WVD. In every case of noisy signals, however, CM of MST was higher than CMs of other non-quadratic methods and CM of MST-masked WVD was also higher than CMs of other masked WVDs. RE of the TFR of signal 2 delivered by MST was lower than TFRs delivered by other non-quadratic methods at each SNR. Also, RE of MST-masked WVD was lower than other masked WVDs at the same SNR.

**Table 3:** Comparisons of CMs of TFRs of the synthesized signal 2.

SNR (dB)	WVD (×10^−3^)	Masking TFR		Masked WVD (×10^−3^)
		Method	CM(×10^−3^)	
Clean	5.4315	STFT	5.4206	10.0342
		ST	4.0433	7.2938
		IGST	**5.5463**	10.1613
		MST	5.5295	**10.2267**
15	5.2256	STFT	4.8668	9.7235
		ST	3.9190	7.1619
		IGST	5.0224	9.9196
		MST	**5.0342**	**9.9854**
10	4.9486	STFT	4.4099	9.0552
		ST	3.8088	6.8548
		IGST	4.5908	9.2922
		MST	**4.6092**	**9.3642**
5	4.5428	STFT	3.8701	7.6886
		ST	3.6612	6.2615
		IGST	4.0523	7.9593
		MST	**4.0596**	**8.0130**

**Table 4:** Comparisons of REs (at *ϵ* = 3) of TFRs of the synthesized signal 2.

SNR (dB)	WVD	Masking TFR		Masked WVD
		Method	RE	
Clean	14.7061	STFT	14.8111	12.7351
		ST	15.7507	13.6833
		IGST	14.7635	12.6786
		MST	**14.7492**	**12.6509**
15	14.8056	STFT	15.0615	12.7854
		ST	15.8146	13.7048
		IGST	14.9789	12.7104
		MST	**14.9590**	**12.6845**
10	14.9652	STFT	15.3161	12.9465
		ST	15.8820	13.8136
		IGST	15.2034	12.8575
		MST	**15.1792**	**12.8290**
5	15.2422	STFT	15.7018	13.3581
		ST	15.9872	14.0753
		IGST	15.5573	13.2459
		MST	**15.5383**	**13.2203**

### Application to ECG signals

**Fig 12:**
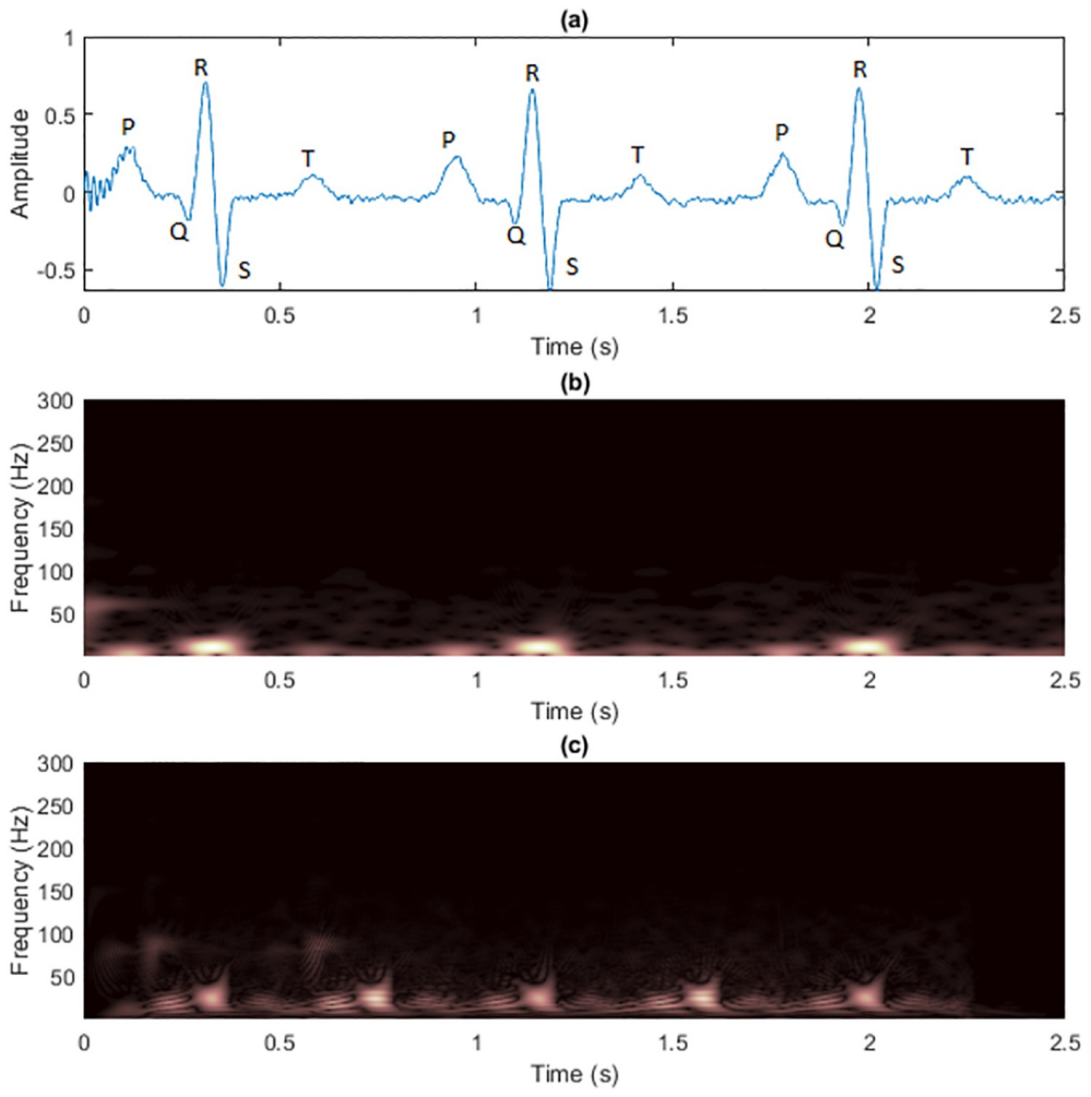
The clean pre-processed ECG signal 1 is shown as (a) time-domain signal, (b) spectrogram and (c) WVD.

**Fig 13:**
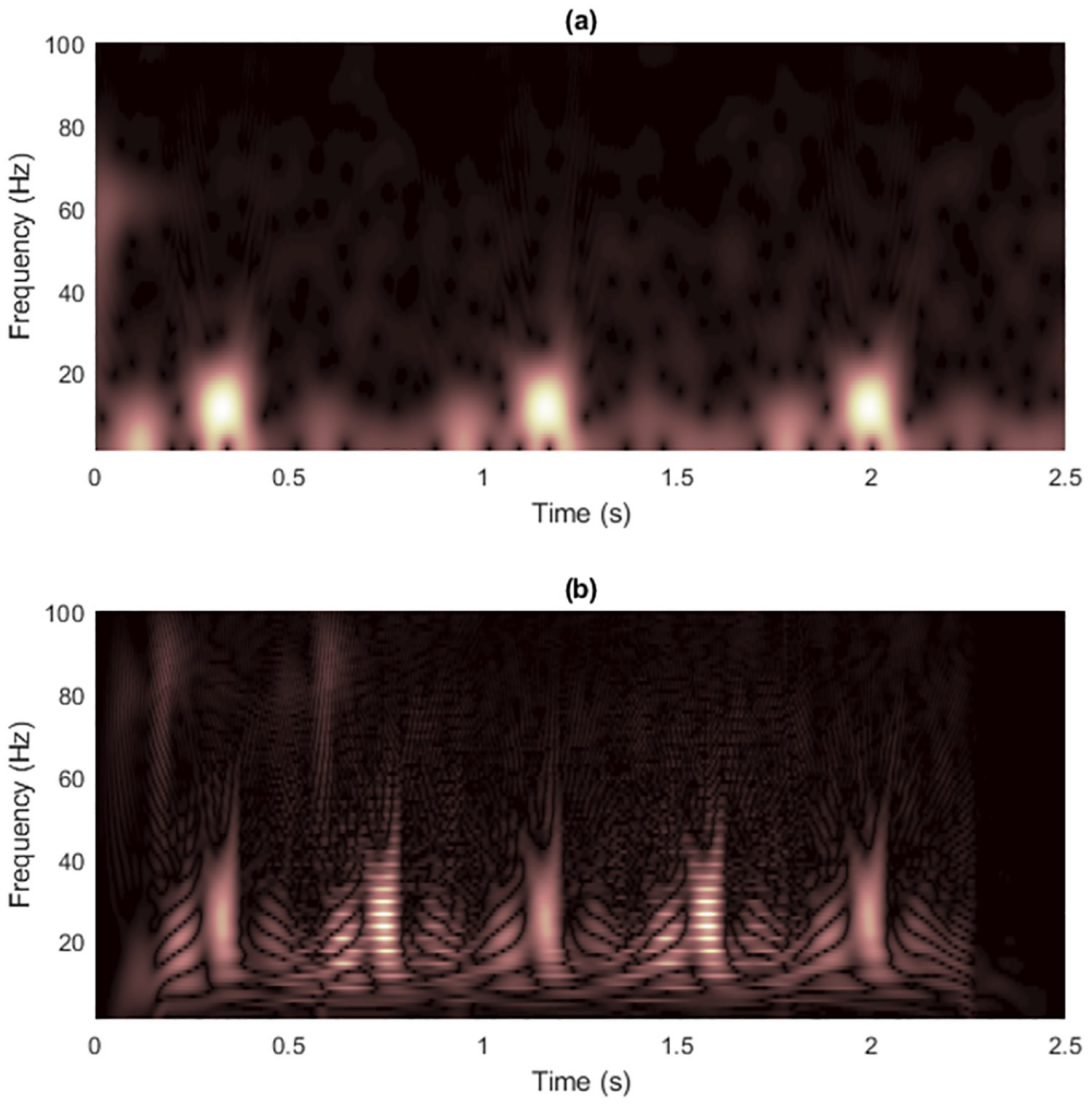
The zoomed clean pre-processed ECG signal 1 is shown as (a) spectrogram and (b) WVD.

Previously, we demonstrated and discussed the advantages of the masking technique to reduce cross-terms in TFRs of the two synthetic signals. This subsection details the application of the technique on real-world electrocardiogram (ECG) signals and shows the experimental results. The selected two ECG signals were first denoised using two kinds of filters. First, 60-Hz, 120-Hz and 180-Hz notch filters were employed to remove the harmonic hum in the power line. Second, a bandpass filter was implemented with passband frequency of 0.25 to 40 Hz to remove the DC drift and muscle noises [59]. The ECG signal 1 used in [59] had *L*_*x*_ = 1, 500 data samples and sampling rate *f*_*s*_ = 600 Hz (Fig 12). The zoomed TFRs focusing on pulses of ECG signal 1 are shown in Fig 13. R waves had the highest amplitudes in time-domain as shown having brightest colors in the TFRs. R waves in the WVD then seemed to be the most robust to cross-terms, while resolution of other ECG waves (P, Q, S and T waves) were severely destroyed.

**Fig 14:**
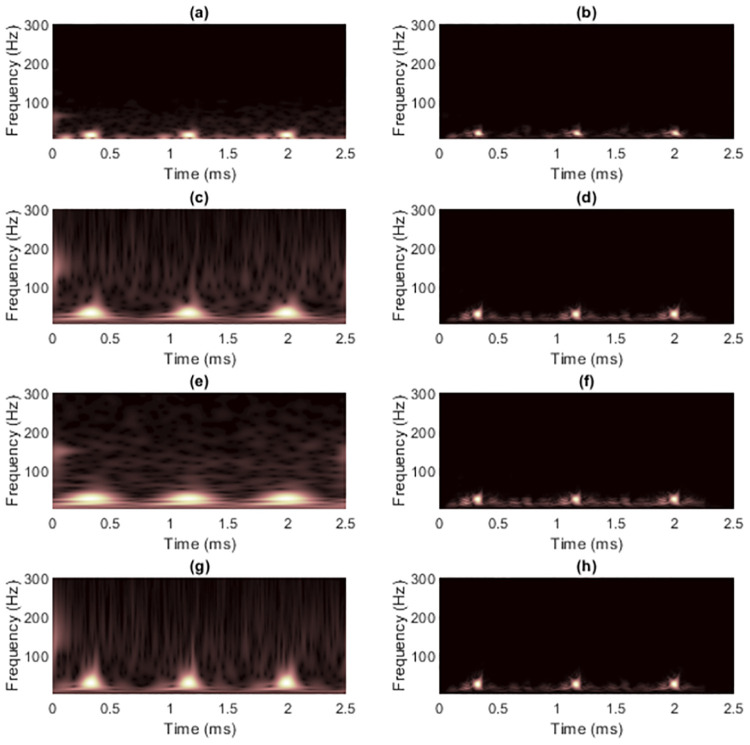
TFRs of the clean pre-processed ECG signal 1 obtained from (a) STFT, (b) the STFT-masked WVD, (c) the original ST [24], (d) the ST-masked WVD, (e) the IGST [51] with *P*_opt_ = {3.0000, 1.5701, 0.5247}, (f) the IGST-masked WVD, (g) the MST [26] with *γ* = 20 and (h) the MST-masked WVD.

**Fig 15:**
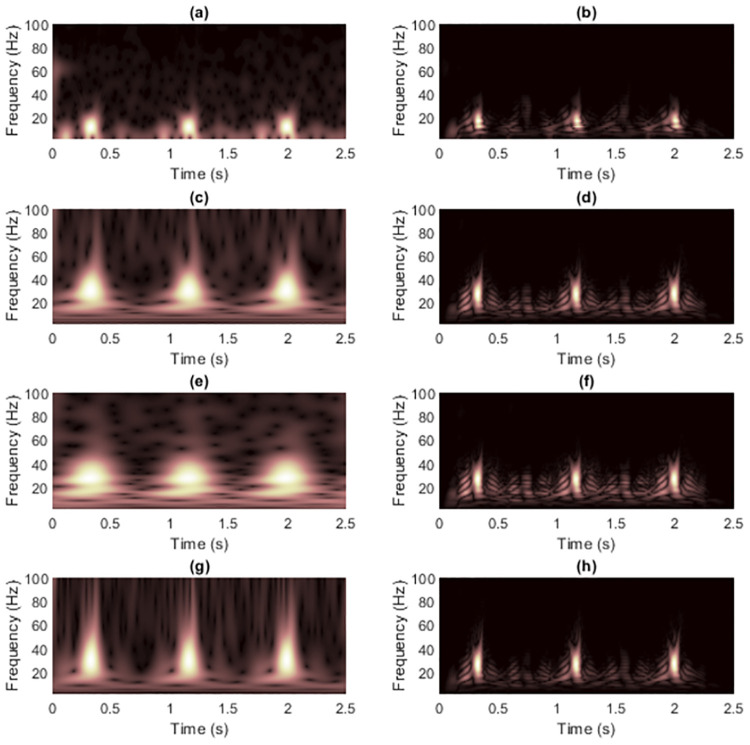
Zoomed version of TFRs in Fig 14.

Fig 14 shows WVD masking results on ECG signal 1, while the zoomed version was shown in Fig 15. Here, we focus on the cropped TFRs at the frequency range of 1-100 Hz because of clearer comparisons. The original ST preserved the peaks of R waves but did not provide adequate quality for the other waves because other waves had low-frequency energies that spread along the time-axis. The IGST, this time, provided the R wave peaks that are more spreading along time-axis as compared to the other STs. The conditions in Eqs 10 and 11 that are used to set the range of allowed parameter values for the window adjusting are proved not universally suitable for any input signal. The MST provided clearer resolutions of the other waves along with the R waves that showed the most peaks compared to the other waves and the pair of wave peaks that are generated next to each of the actual R wave in the both sides is also preserved (as compared to the STFT). Recall that window function used in MST depends on the input signal while the others do not, but the parameter *γ* still needs to be carefully chosen. However, as the WVD had already destroyed the resolutions of the P, Q, S and T waves, WVD masking was unsuccessful in recovering the resolution of these waves in this case but it increased the sharpness of the R waves.

Tables 5 and 6 respectively compares the CMs and REs of the original WVD, TFRs used to mask the WVD, and masked WVDs of the clean pre-processed ECG signal 1 at different matrix dimensions, i.e., cropped TFRs (according to the shown zoomed TFRs in Fig 13) and full-dimension TFRs. This time, concentration of IGST-masked WVD was inferior to other masked WVDs by having lowest CM and highest RE. Trend of CMs were less consistent that REs because, in case of full-dimension TFRs, CM of IGST was higher than CM of original ST but CM of IGST-masked WVD was lower than CM of STFT-masked WVD. CM and RE of TFR delivered by MST were better that those of TFRs delivered by other STs but inferior to only those of STFT. This supported the illustrated results shown in Fig 15.

**Table 5:** Comparisons of CMs of TFRs of the clean pre-processed ECG signal 1.

Dimension	WVD (×10^−3^)	Masking TFR		Masked WVD (×10^−3^)
		Method	CM(×10^−3^)	
1-100 Hz × 1,500 samples	6.3681	STFT	**7.4166**	**13.2533**
		ST	4.6953	10.4992
		IGST	4.3110	9.2718
		MST	4.9144	11.6928
1-300 Hz × 1,500 samples (Full dimension)	6.0200	STFT	**7.1994**	**13.2446**
		ST	3.7024	10.4466
		IGST	3.7363	9.2421
		MST	3.8621	11.6379

**Table 6:** Comparisons of REs (at *ϵ* = 3) of TFRs of the clean pre-processed ECG signal 1.

Dimension	WVD	Masking TFR		Masked WVD
		Method	RE	
1-100 Hz × 1,500 samples	14.1068	STFT	**13.7704**	**12.0338**
		ST	15.1696	12.7307
		IGST	15.4872	13.0829
		MST	14.9892	12.4393
1-300 Hz × 1,500 samples (Full dimension)	14.2293	STFT	**13.8349**	**12.0352**
		ST	15.7184	12.7416
		IGST	15.8118	13.0898
		MST	15.5420	12.4495

**Fig 16:**
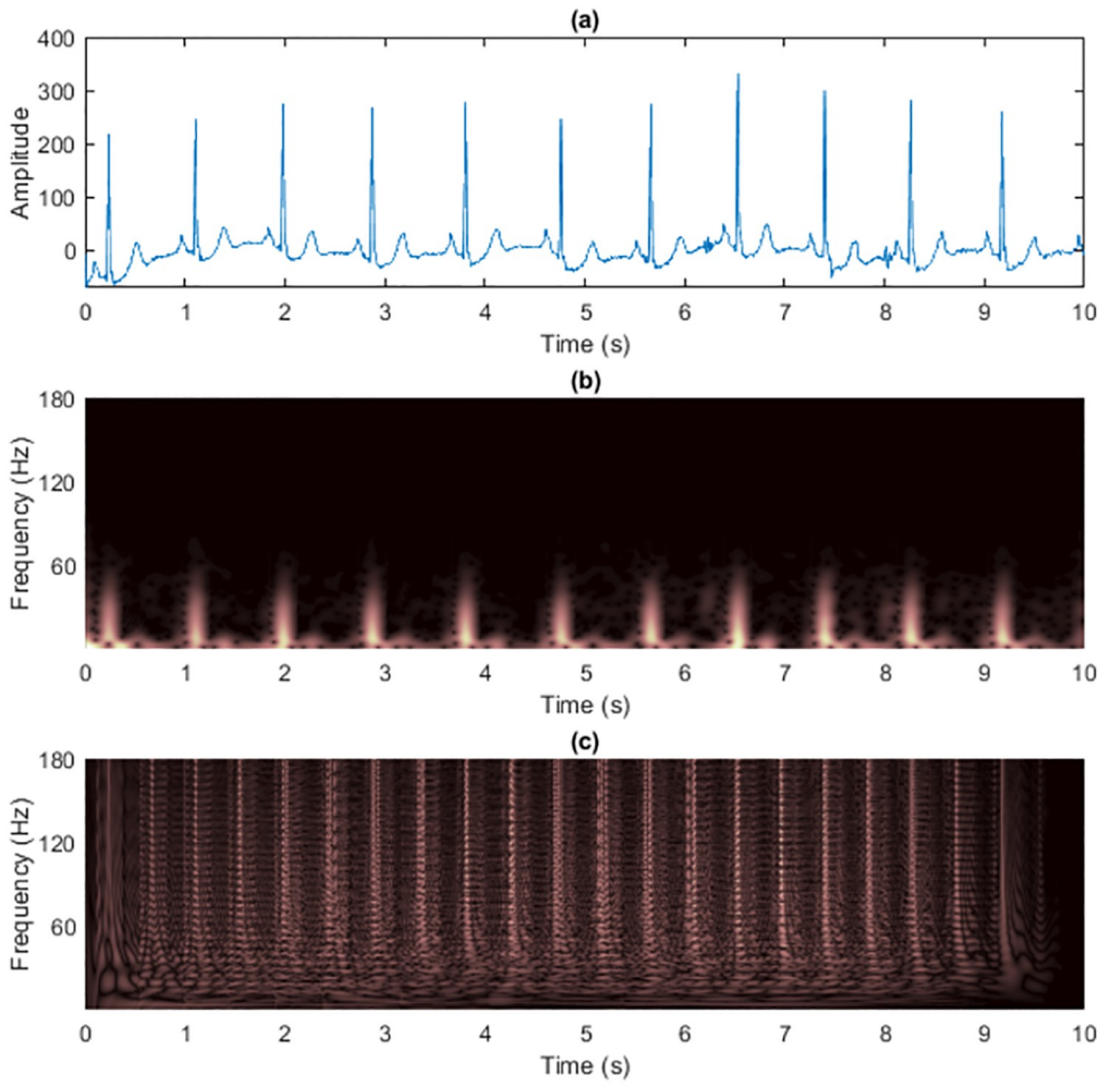
The clean pre-processed ECG signal 2 is shown as (a) time-domain signal, (b) spectrogram and (c) WVD.

**Fig 17:**
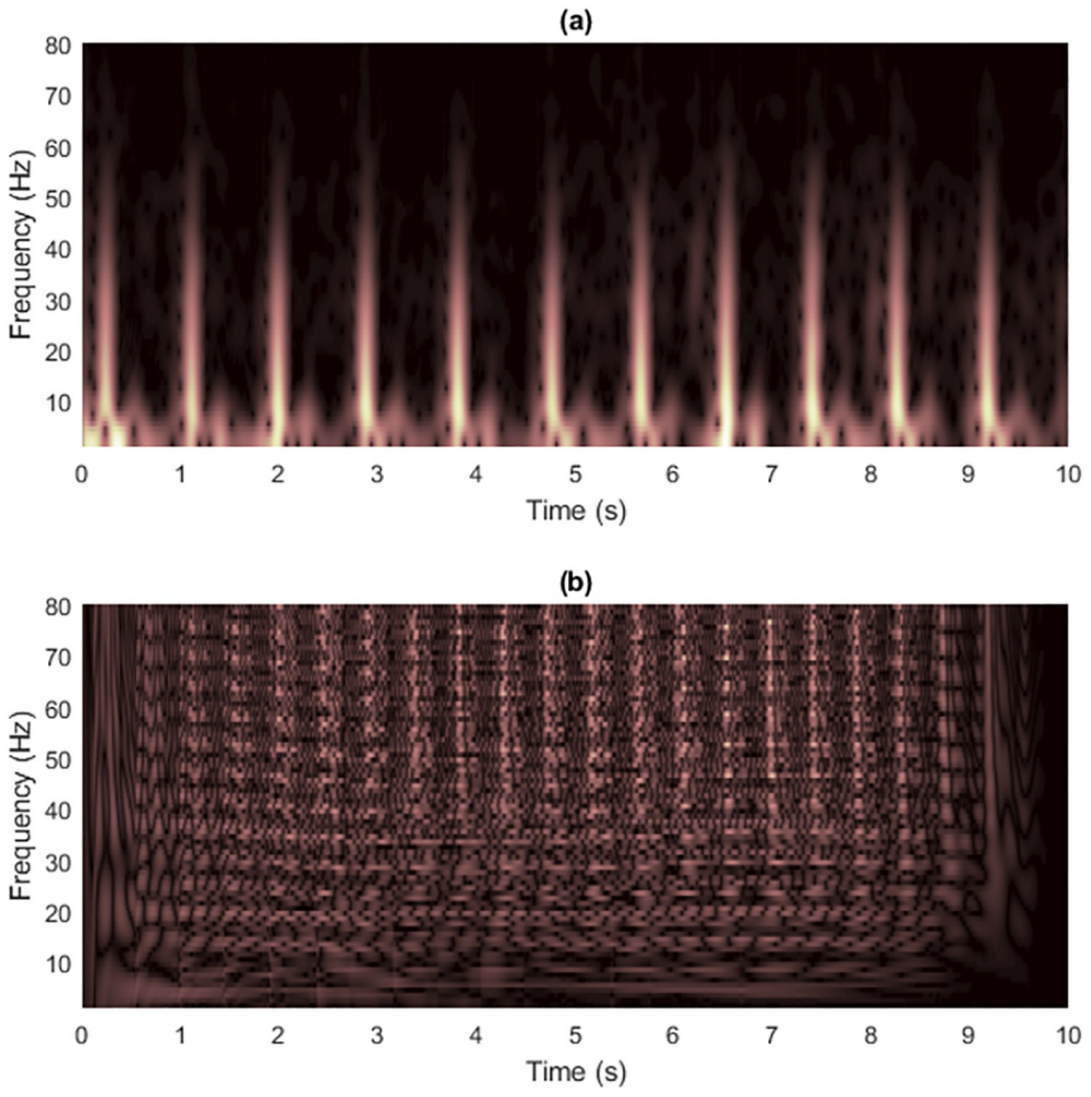
The zoomed clean pre-processed ECG signal 2 is shown as (a) spectrogram and (b) WVD.

We further performed experiments on another ECG signal (called ECG signal 2, shown in Fig 16) retreived from the MIT-BIH Arrhythmia Database [60, 61], *L*_*x*_ = 3, 600 data samples with a sampling rate *f*_*s*_ = 360 Hz. The zoomed TFRs focusing on pulses of ECG signal 2 are shown in Fig 17. The maximum frequency of ECG signals can be up to 250 Hz, so *f*_*s*_ should be much higher in practice [62]. However, this did not matter here because we investigated the effects of masking WVDs with non-quadratic TFRs at the predefined sampling rate.

**Fig 18:**
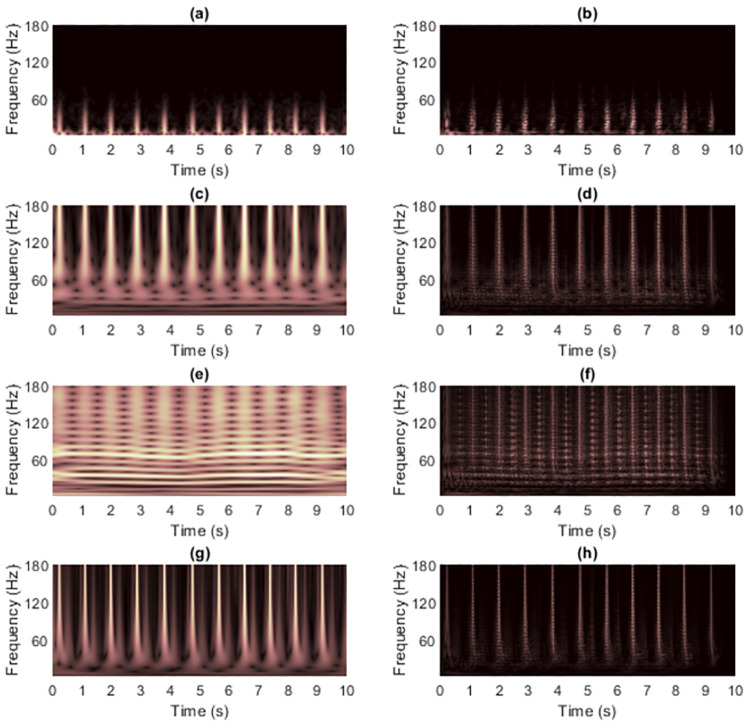
TFRs of the clean pre-processed ECG signal 2 obtained from (a) STFT, (b) the STFT-masked WVD, (c) the original ST [24], (d) the ST-masked WVD, (e) the IGST [51] with *P*_opt_ = {2.2190, 1.4997, 0.5357}, (f) the IGST-masked WVD, (g) the MST [26] with *γ* = 2.5 × 10^−4^ and (h) the MST-masked WVD.

**Fig 19:**
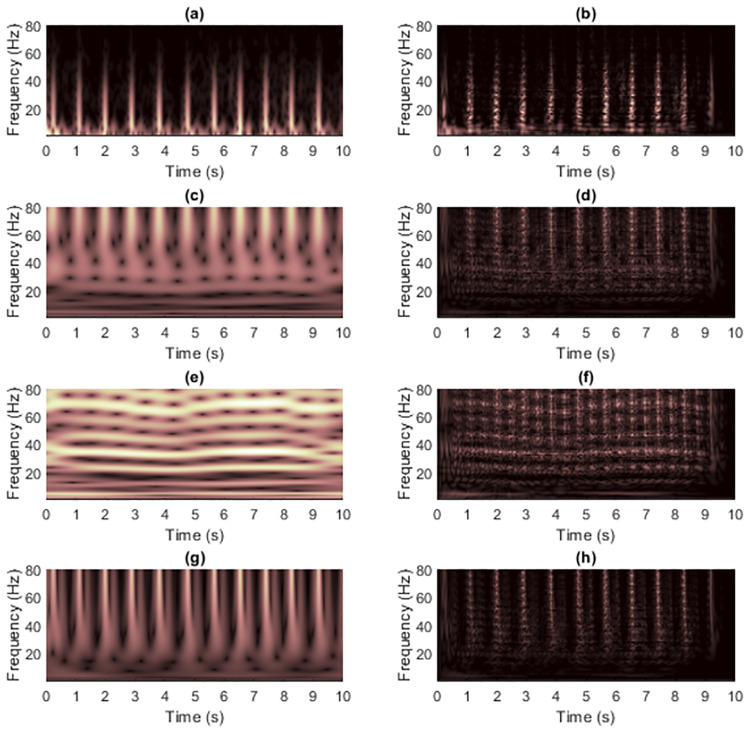
Zoomed version of TFRs in Fig 18.

Fig 18 shows WVD masking results on ECG signal 2, while the zoomed version was shown in Fig 19. The ST [24] and MST [26] yielded sharper resolution of R waves that was less spreading in the time-domain compared to the STFT but they caused the masked R waves to have the maximum frequencies higher than the actual waves (only around 60 Hz) and their TFRs are less comprehensible at the low-frequency regions since the false components occurred. However, the problem in comprehending the low-frequency region is more severe in the original ST when compared to the MST. The reason is that MST applies the window function that depends on the input signal. Consequently, the MST-masked WVD looks better than the ST-masked WVD. The IGST [51], however, delivered the least comprehensible TFR and consequently caused the effectiveness in masking the WVD to be not fruitful. This possibly caused by the conditions (in Eqs 10 and 11) in setting the parameters that may not be suitable for universal applications.

Tables 7 and 8 respectively compares the CMs and REs of the original WVD, TFRs used to mask the WVD and masked WVD of the clean pre-processed ECG signal 2 at different matrix dimensions, i.e., cropped TFRs (according to the zoomed spectrogram in Fig 17(a)) and full TFRs. Concentration of the TFR delivered by IGST was the worst in both terms of CM and RE. IGST-masked WVD also had lowest CM and the highest RE compared to the other masked WVDs. Concentration of MST-masked WVD were only inferior to the STFT-masked WVD. Note that the value of parameter *γ* in MST must be set to be low for this signal, because it is sensitive to time-domain amplitudes that have extremely low and high values, a5s shown in Fig 16(a).

**Table 7:** Comparisons of CMs of TFRs of the clean pre-processed ECG signal 2.

Dimension	WVD (×10^−3^)	Masking TFR		Masked WVD (×10^−3^)
		Method	CM(×10^−3^)	
1-80 Hz × 3,600 samples	2.7338	STFT	**4.5085**	**5.7853**
		ST	2.2517	3.6142
		IGST	2.1238	3.2541
		MST	2.5451	4.6204
1-180 Hz × 3,600 samples (Full dimension)	1.9880	STFT	**4.4636**	**5.7203**
		ST	1.8228	3.2497
		IGST	1.3531	2.2402
		MST	2.1849	4.2497

**Table 8:** Comparisons of REs (at *ϵ* = 3) of TFRs of the clean pre-processed ECG signal 2.

Dimension	WVD	Masking TFR		Masked WVD
		Method	RE	
1-80 Hz × 3,600 samples	16.6993	STFT	**15.3136**	**14.4108**
		ST	17.4222	15.6301
		IGST	17.6592	16.0690
		MST	16.9647	14.8710
1-180 Hz × 3,600 samples (Full dimension)	17.5457	STFT	**15.3353**	**14.4353**
		ST	17.9938	15.9541
		IGST	18.9829	17.1185
		MST	17.3336	15.1893

## Section 5: Discussion

Modification on parameters controlling window length of original ST improved resolution of high-frequency energies of ST-delivered TFR of most of selected signals. Cross-terms located in WVDs of synthetic signals could be masked more effectively in case modified STs were employed. Resolution of low-frequency energies, however, still were not enhanced well. As could be seen in case of ECG signals, the true energies of the ECG signals were supposed to be located at region of low-frequency energies in TFRs. Consequently, low-frequency energies of ST-masked WVDs of ECG signals could not be obtained with sufficiently high resolution.

CM was widely used to measure concentration of TFRs. However, according to the obtained results, the non-quadratic TFR with highest CM did not always deliver CM the masked WVD with highest CM. RE was another metric employed to investigate the results of WVD masking. REs of TFRs had a more consistent trend where the non-quadratic TFR with lowset RE delivered the masked WVD with RE that was lower than REs of the other masked WVDs. With any employed non-quadratic method, masking technique was proved increasing CM and decreasing RE of the original unmasked WVD at every SNR. Nevertheless, despite the improvement of resolutions in terms of CMs and REs, there was no clear universal relationships between these quantities and readability of TFRs.

## Section 6: Conclusions

This paper presents a pilot study as an analysis and simulation comparing the original ST and modified STs to suppress cross-terms in WVDs as performed in WVD masking that originally used STFT. STs that are employed to mask WVDs of non-stationary signals should first be modified with additional parameters to obtain TFRs with better concentration proved with higher energy CMs and REs. These TFRs can then be used to mask WVDs to obtain new TFRs with suppressed cross-terms and, consequently, achieve better resolution. Illustrated in our experiments that suitable optimization technique needs to be incorporated in order to successfully suppress cross-terms for all non-stationary signals for the problem at hands. However, numerical metrics (i.e., energy CMs and REs) of any TFR cannot be used to guarantee readability of the TFR that must be further verified by experts. Each modified ST algorithm may be optimal for some specific problems with parameters that must be employed and configured by experts. Also, there still can be rooms for future work that focuses on more effective cross-term reduction techniques.
